# Immediate complete revascularization showed better outcome in out-of-hospital cardiac arrest survivors with left main or triple-vessel coronary diseases

**DOI:** 10.1038/s41598-022-08383-x

**Published:** 2022-03-14

**Authors:** Youn-Jung Kim, Duk-Woo Park, Yong Hwan Kim, Minwoo Choi, Su Jin Kim, Gun Tak Lee, Dong Hun Lee, Byung Kook Lee, Joo Suk Oh, Sang Hoon Oh, Dong Hoon Lee, Won Young Kim

**Affiliations:** 1 Department of Emergency Medicine, Asan Medical Center, University of Ulsan College of Medicine, 88, Olympic-ro 43-gil, Songpa-gu, Seoul, 05505 Republic of Korea; 2 Department of Cardiology, Asan Medical Center, University of Ulsan College of Medicine, Seoul, Republic of Korea; 3 Department of Emergency Medicine, Samsung Changwon Hospital, Sungkyunkwan University School of Medicine, Changwon, Republic of Korea; 4 Department of Emergency Medicine, Yonsei University Severance Hospital, Seoul, Republic of Korea; 5 Department of Emergency Medicine, Korea University College of Medicine, Seoul, Republic of Korea; 6 Department of Emergency Medicine, Samsung Medical Center, Sungkyunkwan University School of Medicine, Seoul, Republic of Korea; 7 Department of Emergency Medicine, Chonnam National University Medical School, Gwangju, Republic of Korea; 8 Department of Emergency Medicine, Uijeongbu St. Mary’s Hospital, College of Medicine, The Catholic University of Korea, Uijeongbu-si, Republic of Korea; 9 Department of Emergency Medicine, Seoul St. Mary’s Hospital, College of Medicine, The Catholic University of Korea, Seoul, Republic of Korea; 10 Department of Emergency Medicine, Chung-Ang University, College of Medicine, Seoul, Republic of Korea

**Keywords:** Cardiology, Medical research

## Abstract

This study aimed to evaluate the prevalence of left main or triple vessel coronary artery disease (CAD) in comatose out-of-hospital cardiac arrest (OHCA) survivors and assessed their outcome based on the revascularization strategy. This multicenter, retrospective, observational registry-based study was conducted at 9 Korean tertiary care hospitals. Adult comatose OHCA survivors with left main or triple vessel CAD documented by immediate (≤ 2 h) coronary angiography after return of spontaneous circulation between 2011 and 2019 were included. The primary outcome was neurologically intact survival at 1-month. Among 727 OHCA patients, 150 (25%) had left main or triple vessel CAD and underwent complete (N = 32), incomplete (N = 78), and no immediate (N = 40) revascularization, respectively. The rate of neurologically intact survival at 1 month was significantly different among the groups (53%, 32%, and 23% for complete, incomplete, and no immediate revascularization groups, respectively; P = 0.02). After adjustment using the inverse probability of treatment weighting, complete revascularization was associated with neurologically intact survival at 1 month (odds ratio, 2.635; P = 0.01). Left main or triple vessel CAD is not uncommon in OHCA patients. The complete revascularization was associated with better outcome. Further clinical trials to confirm the best revascularization strategy are needed.

## Introduction

Out-of-hospital cardiac arrest (OHCA) is a leading cause of global mortality and morbidity, and coronary artery disease (CAD) is the most common underlying cause of OHCA^1^. Among cardiac arrest survivors, 40%-70% had obstructive CAD, and more than 50% had multivessel obstructive CAD, which requires percutaneous coronary intervention (PCI)^2,3^. Indeed, multivessel CAD is highly likely to induce widespread myocardial ischemia and progressive left ventricular dysfunction, and it has thus been associated with increased mortality^2,4,5^. Previous guidelines recommended complete revascularization for patients with cardiogenic shock because of theoretical benefits such as salvage of the myocardium, improvement in circulatory function, and prevention of additional major adverse cardiac events^2,5,6^. However, a recent trial demonstrated that a culprit lesion-only strategy was superior to immediate multivessel PCI for patients with cardiogenic shock and multivessel CAD^6,7^.

While multivessel CAD is frequently observed in survivors of cardiac arrest, past studies have not accounted for differences in the management strategy of those with multivessel CAD, and current guidelines also do not address this issue. Postcardiac arrest syndrome (i.e., systemic ischemia/reperfusion damage including hypoxic-ischemic brain injury and myocardial dysfunction) in OHCA survivors differ from cardiogenic shock^8–10^. Moreover OHCA survivors with multivessel CAD tend to be refractory to resuscitative effort and have hemodynamic instability after the return of spontaneous circulation; consequently, such patients constitute a more complex. In this study, we evaluated the prevalence of left main or triple vessel CAD in comatose OHCA survivors and assessed their outcome based on the revascularization strategy.

## Results

**Figure 1:**
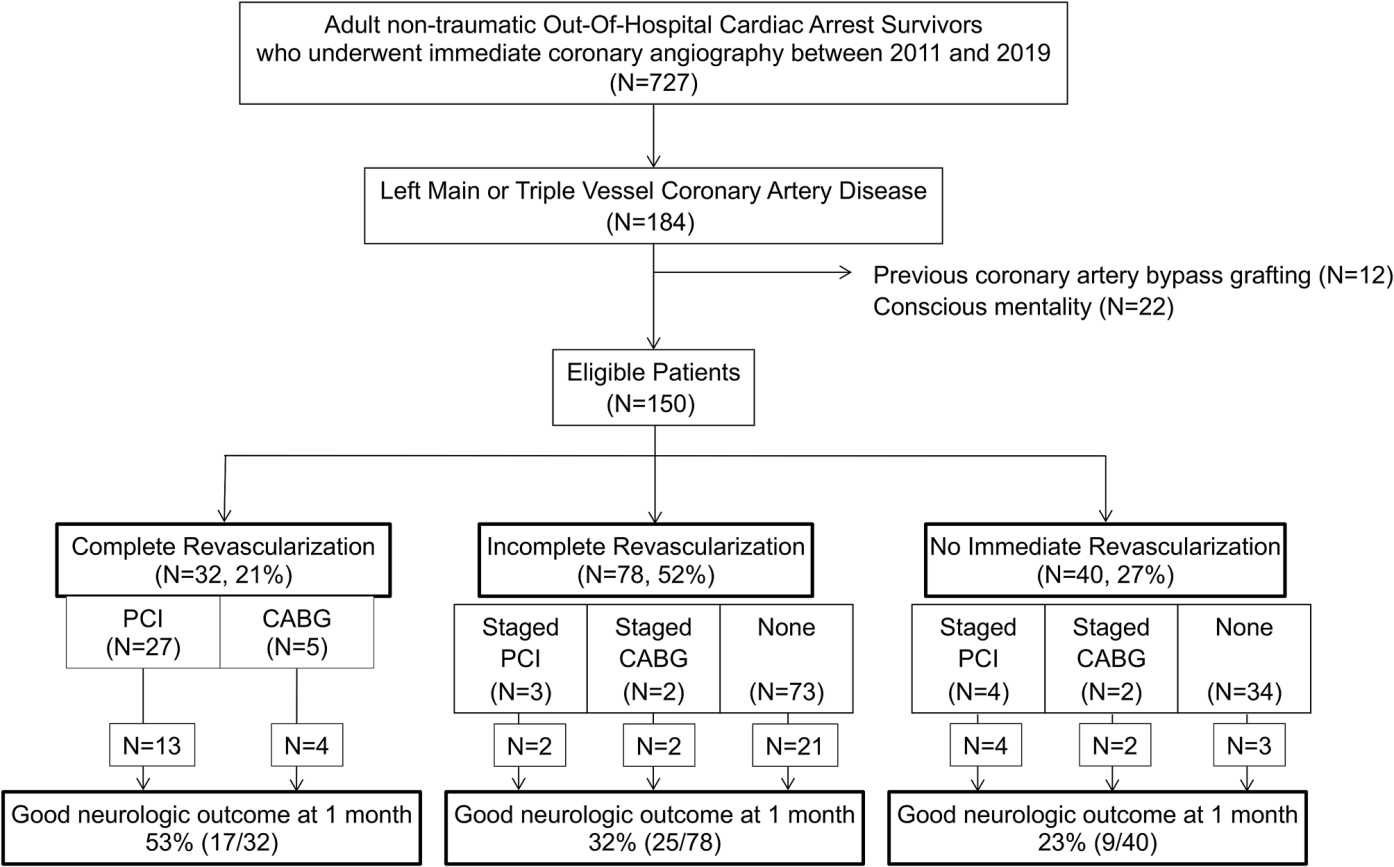
Flow diagram of the patient selection process. *CABG* coronary artery bypass grafting, *PCI* percutaneous coronary intervention.

Out of the 727 patients with non-traumatic OHCA who received immediate coronary angiography during the study period, 184 were diagnosed with left main or triple vessel CAD and enrolled in this study; of them, 34 patients were excluded due to previous history of CABG (N = 12) or consciousness (N = 22). Therefore, a total of 150 patients (complete revascularization group, N = 32; incomplete revascularization group, N = 78; no immediate revascularization group, N = 40) were finally included in the analysis (Fig. 1). The prevalence of left main or triple vessel CAD in comatose OHCA survivors was 25.3%, and the rate of neurologically intact survival at 1-month was 34%. Patients in the complete revascularization group received either PCI (N = 27) or immediate CABG (N = 5; Supplementary Table S1 online). Inverse probability of treatment weighting (IPTW) analysis was performed between the complete revascularization group (N = 32) and the incomplete/no immediate revascularization group (N = 118).

The demographic and clinical characteristics of the patients according to the revascularization strategies are summarized in Table 1. There were no significant intergroup differences in the demographic and clinical features including age, sex, diabetes, and resuscitation profiles. The proportion of chronic kidney disease was significantly different among the three groups (p = 0.001).

**Table 1:** Baseline characteristics of patients according to revascularization strategies.

Characteristics	Total (n = 150)	Complete (n = 32)	Incomplete (n = 78)	No immediate (n = 40)	*P*-value
Age, years	63.5 (54.0–71.0)	61.5 (52.0–68.8)	65.5 (55.8–71.0)	63.0 (53.3–76.8)	0.38
Male sex	129 (86%)	31 (97%)	65 (83%)	33 (83%)	0.14
**Comorbid disease**					
Hypertension	81 (54%)	16 (50%)	46 (59%)	19 (48%)	0.44
Diabetes mellitus	61 (41%)	11 (34%)	32 (41%)	18 (45%)	0.66
Chronic kidney disease	10 (7%)	1 (3%)	1 (1%)	8 (20%)	0.001
Previous stroke	14 (9%)	2 (6%)	8 (10%)	4 (10%)	0.87
Previous PCI	19 (13%)	3 (9%)	10 (13%)	6 (15%)	0.77
**Arrest characteristics**					
Witnessed	124 (83%)	26 (81%)	64 (82%)	34 (85%)	0.90
Bystander CPR	92 (61%)	20 (63%)	47 (60%)	25 (63%)	0.96
Initial shockable rhythm	86 (57%)	16 (50%)	47 (60%)	23 (58%)	0.61
No flow time, min	2.0 (0.0–5.0)	1.0 (0.0–5.0)	2.0 (0.0–5.0)	1.0 (0.0–6.0)	0.73
Total resuscitation duration, min	27.5 (13.0–41.5)	28.5 (12.3–44.8)	25.0 (12.0–43.0)	26.5 (17.5–37.3)	0.94
Total resuscitation duration ≥ 30 min	68 (45%)	16 (40%)	38 (49%)	14 (44%)	0.66
ST-segment elevation on immediate ECG	78 (52%)	15 (47%)	42 (54%)	21 (53%)	0.82
**Intervention before and during CAG**					
Vasopressor use	95 (63%)	17 (53%)	53 (68%)	25 (63%)	0.34
Extracorporeal life support	49 (33%)	16 (50%)	22 (28%)	11 (28%)	0.06

Values are presented as median (interquartile range) or number (percentage).

*CAG* coronary angiography, *CPR* cardiopulmonary resuscitation, *ECG* electrocardiogram, *PCI* percutaneous coronary intervention.

Table 2 shows the baseline lesion characteristics and subsequent management of the study patients according to the revascularization strategies. The mean time from presentation to the emergency department or the return of spontaneous circulation to coronary angiography was 71.6 min in the overall study population, and there was no significant intergroup difference. The lesion and extent of the coronary artery disease and the proportion of chronic total occlusion differed significantly among the groups. The CAD extent were more complex in patients in the incomplete or no immediate revascularization groups than in those in the complete revascularization group, but the SYNTAX score indicating the anatomical complexity of CAD did not show significant differences among the groups (p = 0.48). Staged PCI was performed in 4% of patients in the incomplete revascularization group and in 10% of them in the no immediate revascularization group (p = 0.11), and elective CABG was performed in 3% of patients in the incomplete revascularization group and 5% of them in the no immediate revascularization group (p = 0.54).

**Table 2:** Coronary angiographic findings and clinical outcomes according to revascularization strategies.

Variables	Total (n = 150)	Complete (n = 32)	Incomplete (n = 78)	No immediate (n = 40)	*P*-value
Interval from ROSC to CAG, min	71.6 (38.5)	66.6 (38.1)	72.5 (37.7)	73.8 (41.0)	0.71
**Coronary stenosis**					
Left main artery	58 (398.7%)	21 (65.6%)	22 (28.2%)	15 (387.5%)	0.001
Left anterior descending artery	138 (92.0%)	25 (78.81%)	75 (96.2%)	38 (95.0%)	0.008
Left circumflex artery	132 (88.0%)	19 (59.4%)	75 (96.2%)	38 (95.0%)	< 0.001
Right coronary artery	130 (86.7%)	18 (56.3%)	74 (954.9%)	38 (95.0%)	< 0.001
**Disease extent**					< 0.001
Left main artery only	5 (3.3%)	4 (132.5%)	0 (0%)	1 (32.5%)	
Left main artery plus 1-vessel disease	10 (6.7%)	8 (25.0%)	1 (1.3%)	1 (32.5%)	
Left main artery plus 2-vessel disease	15 (10.0%)	5 (15.6%)	9 (121.5%)	1 (32.5%)	
Left main artery plus 3-vessel disease	28 (198.7%)	4 (132.5%)	12 (15.4%)	12 (30.0%)	
Triple vessel disease	92 (61.3%)	11 (34.4%)	56 (721.8%)	25 (632.5%)	
SYNTAX Score	29.5 (23.0–38.1)	28.0 (19.4–42.3)	28.8 (22.4–36.6)	31.8 (25.0–38.1)	0.48
**Vessel related to the infarction**					
Left main artery	35 (23.3%)	18 (56.3%)	16 (210.5%)	1 (32.5%)	< 0.001
Left anterior descending artery	66 (44.0%)	22 (698.8%)	36 (46.2%)	8 (20.0%)	< 0.001
Left circumflex artery	30 (20.0%)	4 (132.5%)	20 (25.6%)	6 (15.0%)	0.19
Right coronary artery	38 (25.3%)	9 (28.1%)	20 (25.6%)	9 (232.5%)	0.86
Chronic total occlusion ≥ 1	69 (46.0%)	6 (198.8%)	41 (532.6%)	22 (55.0%)	0.002
IABP insertion	12 (8.0%)	0 (0%)	9 (121.5%)	3 (87.5%)	0.13
Staged PCI	7 (54.7%)	0 (0%)	3 (43.8%)	4 (10.0%)	0.11
Staged CABG	4 (32.7%)	0 (0%)	2 (32.6%)	2 (5.0%)	0.54
Targeted temperature management	101 (67.3%)	18 (56.3%)	57 (73.1%)	26 (65.0%)	0.22
Survival at 1-month	75 (50.0%)	20 (632.5%)	38 (498.7%)	17 (432.5%)	0.23
Neurologically intact survival at 1-month	51 (34.0%)	17 (53.1%)	25 (32.1%)	9 (232.5%)	0.02

Values are presented as mean (standard deviation), median (interquartile range) or number (percentage).

*CABG* coronary artery bypass grafting, *CAG* coronary angiography, *IABP* intra-aortic balloon pump, *PCI* percutaneous coronary intervention, *ROSC* return of spontaneous circulation.

**Figure 2:**
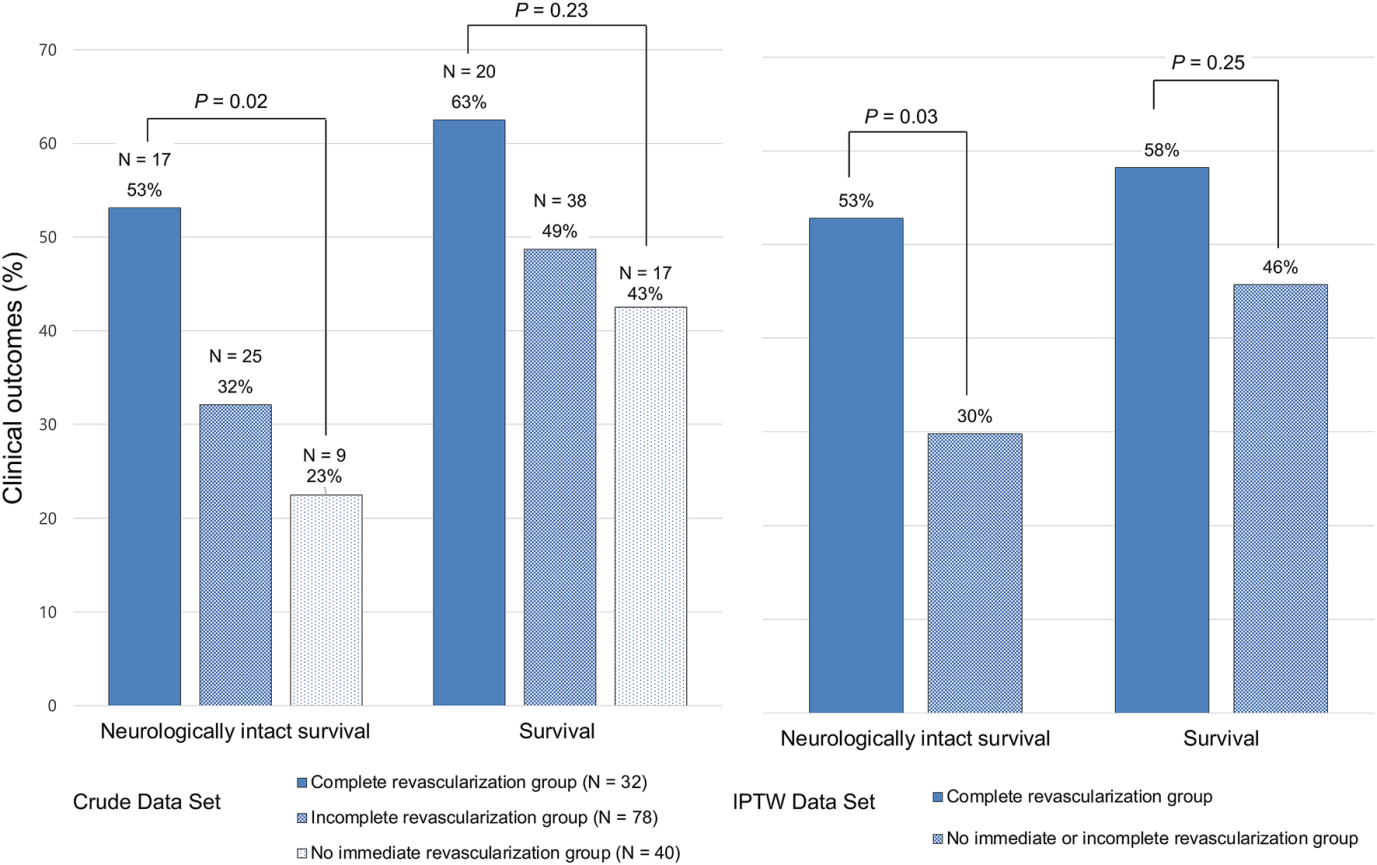
Neurologically intact survival and survival of patients at 1 month according to the revascularization strategies. Crude and adjusted data using the inverse probability of treatment weighting method are shown. *IPTW* inverse probability of treatment weighting.

After adjustment with IPTW for patients in the complete revascularization and those in the incomplete or no immediate revascularization groups, the clinical characteristics and baseline lesion were well balanced between the groups except for sex (Supplementary Table S2 online). Figure 2 shows the comparison of outcomes at 1 month for the unadjusted crude data and the IPTW-adjusted data set. The overall rate of neurologically intact survival and survival at 1 month were 34% and 50%, respectively. In the crude data set, the rate of neurologically intact survival was significantly different among the groups (p = 0.02), with the complete revascularization group having the highest rate (53%) and the no immediate revascularization group having the lowest rate (23%); the rate of survival was not significantly different among the three groups (p = 0.23). After adjustment with IPTW, the rate of neurologically intact survival remained significantly different between the complete revascularization group and the no immediate or incomplete revascularization group (53% vs. 30%; p = 0.03).

The results of the univariable logistic regression analysis for neurologically intact survival and survival at 1 month are presented in Supplementary Tables S3 and S4 online, respectively. The adjusted odds ratios (AORs) for neurologically intact survival and survival at 1 month according to the revascularization strategy are presented in Table 3. Complete revascularization was consistently associated with a higher rate of neurologically intact survival at 1 month in both the crude data set (AOR, 5.997; 95% confidence interval, 1.644–21.878; p = 0.007), and the IPTW-adjusted data set (odds ratio [OR], 2.635; 95% confidence interval, 1.128–6.155; p = 0.01).

**Table 3:** Adjusted odds ratio for neurologically intact survival and survival at 1 month of revascularization strategy.

Model	Revascularization strategy	Neurologically intact survival		Survival	
		OR (95% CI)	*P*-value	OR (95% CI)	*P*-value
Crude	No immediate	Reference	0.02	Reference	0.16
	Incomplete	1.953 (0.694–5.497)	0.21	1.619 (0.647–4.048)	0.30
	Complete	5.997 (1.644–21.878)	0.007	3.187 (0.980–10.361)	0.05
IPTW	No immediate or incomplete	Reference		Reference	
	Complete	2.635 (1.128–6.155)	0.01	1.656 (0.713–3.847)	0.03

*CI* confidence interval, *IPTW* inverse probability of treatment weighting, *OR* odds ratio.

## Discussion

This study evaluated the prevalence of left main or triple vessel CAD using a multicenter registry of comatose OHCA survivors and assessed their outcome based on the revascularization strategy. We found that left main or triple vessel CAD was seen in 25% of comatose OHCA patients and that the rate of neurologically intact survival at 1 month was 34%. Using crude and IPTW analysis suggested that compared with no immediate or incomplete revascularization, complete revascularization is associated with neurologically intact survival in OHCA patients with left main or triple vessel CAD. Also, the IPTW analysis showed that complete revascularization is associated with better 1-month survival compared with no immediate or incomplete revascularization.

Our study is the first to specifically report the role of revascularization strategy in resuscitated OHCA patients with multivessel CAD. Our findings are in contrast with those of a recent trial on patients with multivessel CAD and cardiogenic shock that showed a superior survival benefit of culprit-lesion-only PCI over immediate multivessel PCI^6,7^. However, more recent clinical trials including the patients with ST-segment elevation myocardial infarction and left main or triple vessel CAD demonstrated more favorable long-term outcome of complete revascularization compared to culprit-lesion-only PCI^11,12^. This discrepancy in the results may be due to differences in the patient population, primary endpoint, and the revascularization strategy. OHCA patients with multivessel CAD tend to be a more complex and higher-risk group owing to the prolonged systemic ischemia/reperfusion damage, including hypoxic-ischemic brain injury and myocardial dysfunction^9,10^. Further clinical trials including the OHCA patients with multivessel CAD are needed to confirm the best revascularization strategy.

In our study, the overall rates of neurologically intact survival and survival at 1 month were 34% and 50%, respectively, which were worse than about 60% short-term survival rate reported in recent trials on patients with OHCA or cardiogenic shock with multivessel CAD^5,6^. Only the complete revascularization group in our study showed a comparable 1-month survival rate of 63% and a neurologically intact survival rate of 53%. The complete revascularization strategy had a more favorable effect on neurologic outcomes than on survival. After early stabilization in OHCA survivors, the withdrawal of life-sustaining therapy owing to perceived unfavorable neurological prognosis is the leading cause of death in OHCA patients^13,14^, which suggests that neurologically intact survival should be considered as the primary therapeutic endpoint for comatose OHCA patients. A recent study suggested that the prolonged procedural duration and a higher dose of contrast materials outweigh the potential benefit of myocardial salvage in immediate multivessel PCI for patients with cardiogenic shock with multivessel CAD^6^. Although we provided statistically unbiased results using IPTW analysis, it should be noted that the complete revascularization group in our study had a significantly less complex CAD in terms of disease extent and the presence of chronic total occlusion (19%), which implies that procedural duration and the amount of contrast material in the complete revascularization group might not have been significantly different from those in the no immediate or incomplete revascularization group. The left main-only CAD accounted for 13% of the complete revascularization group, and there was an overlap between the complete revascularization group and culprit-lesion-only PCI. Moreover, chronic total occlusion was the main obstacle for achieving complete revascularization. Owing to the observational nature of our study, we categorized the patients based on the results of the revascularization regardless of the recanalization attempts. However, these are only speculative because the specific data on the procedure progress records and the amount of contrast material were not obtained in this study. It should be noticed that extracorporeal life support using the extracorporeal membrane oxygenation (ECMO) was performed for the half of the patients in the complete revascularization group. Despite the conflicting results of the use of ECMO in OHCA patients, the potential therapeutic role of ECMO that helps preserve organ perfusion might affect the better outcome in complete revascularization group^15^. Also, the possibility of unknown confounders affecting the decision of immediate CABG should be considered when interpreting our results.

Primary PCI strategy is recommended for OHCA patients with suspected cardiac causes^16,17^, but there are no specific guidelines for OHCA patients with CAD who are not amenable to PCI. Furthermore, recent guidelines recommend CABG over PCI for patients with stable left main CAD with high anatomical complexity or stable multivessel CAD with intermediate-to-high anatomical complexity^17,18^, but they do not suggest a specific type of revascularization for patients with left main or multivessel CAD with cardiogenic shock^4^. The demanding and resource-intensive nature of CABG and a tendency for therapeutic nihilism in the comatose OHCA patients contribute to the low rates of CABG surgery as a revascularization strategy; consequently, the potential benefit of CABG remains unclear^4,19^. In our study, the revascularization strategy was based on the individual decision of the interventional cardiologists and cardiac surgeons. Among the 32 (21%) patients in the complete revascularization group, 5 (16%) OHCA patients with CAD not amenable to PCI received immediate CABG, and 4 of those patients showed neurologic recovery after the surgery. Due to the limited number of patients and unknown confounders, our results regarding the positive effect of immediate CABG for comatose OHCA patients with left main or triple vessel CAD should be regarded as hypothesis-generating and highlighting the need for further research. Our results suggest that for incomplete or no immediate revascularization strategy with an absolute risk reduction of 24% for a neurologically intact survival, the number needed to treat would be 4.2.

This study has several limitations. First, as this was an observational study, our results should be considered as showing an independent association between revascularization and neurologically intact survival and not a causal relationship thereof. Second, the technical and medical advances in cardiovascular and post-resuscitation care during the study period should be considered. Since the guidelines have been updated, subsequent interventions and treatment strategies should have been applied differently during the study period, which could have affected the clinical outcomes and acted as potential confounding factors. Third, the small sample size limits the internal and external validity of the study results, and our results were underpowered for detecting significant differences in the clinical outcomes between the no immediate group and the incomplete revascularization group. Larger studies are needed to conclusively confirm these findings. Moreover, post-resuscitation care for OHCA patients involves a complex series of clinical decisions and could have acted as a potential confounding factor. Fourth, although we adjusted for confounding clinical covariates using IPTW analysis, the intergroup differences may have been due to other unknown confounders. Lastly, the patients in complete revascularization group had significantly less complex CAD extent. Although the SYNTAX scores did not differ significantly among the groups, the score tended to be lower in the complete revascularization group (median, 28.0; interquartile ranges, 19.4–42.3) compared to the no immediate revascularization group (median, 31.8; interquartile ranges, 25.0–38.1). These differences would affect the prognosis in patients with OHCA despite the statistical adjustment with IPTW.

In summary, approximately one-fourth of OHCA survivors had left main or triple vessel CAD. Their neurologically intact survival at 1 month was 34%, and it was greater in patients after complete revascularization than in patients with incomplete or no immediate revascularization. We found that complete upfront revascularization was associated with better neurologic outcomes, and this will be helpful for physicians while making difficult decisions regarding the appropriate revascularization strategy in resuscitated patients with multivessel CAD. We believe that our study has implications for future clinical trials on revascularization strategy.

## Methods

### Study design and patients

This multicenter, retrospective, registry-based observational study was conducted at the emergency departments of nine tertiary care university-affiliated teaching hospitals in the Republic of Korea. The OHCA registries of each center enrolled consecutive adult (aged ≥ 18 years) patients with non-traumatic OHCA who were transported to the participating emergency departments with resuscitation efforts^20,21^. The Institutional Review Board of the University of Ulsan College of Medicine reviewed and approved the study protocol (No. 2015–1224) and waived the need for informed consent considering the retrospective nature of the study. The study has been performed in accordance with the ethical standards laid down in the 1964 Declaration of Helsinki and its later amendments.

Patients were included when they had no obvious extracardiac cause of OHCA, such as hanging, drowning, asphyxia, and poisoning; were unconscious after the return of spontaneous circulation, defined as Glasgow Coma Scale of 8 or less; and were documented with left main or triple vessel CAD with > 50% stenosis on coronary angiography performed within 2 h after the return of spontaneous circulation or presentation to the emergency department between January 2011 and December 2019. Coronary angiography was performed in OHCA patients with ST-segment elevation on electrocardiography and in patients without ST-segment elevation with suspected cardiac origins^22,23^. We excluded patients when they met the following conditions: those with terminal illness documented in medical records; those under hospice care; pregnant women; those with a pre-documented “Do Not Resuscitate” card; and those with prior CABG.

### Management and data collection

During the study period, all OHCA patients received cardiopulmonary resuscitation and post-resuscitation care in accordance with the standard intensive care protocols at each institution. The decision for PCI, CABG, or medical treatment after coronary angiography was based on the judgment of the interventional cardiologist and the angiographic findings. Anticoagulant agents were administered according to standard regimens, and glycoprotein IIb/IIIa inhibitors were administered at the discretion of the physician.

Demographic and clinical data, including those on age, sex, comorbid diseases, resuscitation profiles, and interventions at the emergency department, were extracted from the OHCA registries. To determine the survival status and the neurologic status according to the Cerebral Performance Category score, clinical follow-up was conducted at 1 month by the investigators at each participating center through standardized follow-up telephone interviews with the patient or a primary caregiver (family member). Coronary angiographic findings and the interval from presentation to the emergency department to coronary angiography were also retrieved from the electronic medical records. The investigators calculated The SYNTAX scores using dedicated software (available at http://syntaxscore.org) to present the anatomical complexity of CAD.

Patients were categorized into three groups according to their initial revascularization treatment based on anatomical parameters: (1) no immediate revascularization with the option of staged revascularization; (2) incomplete revascularization, defined that the criteria for anatomic complete revascularization were not achieved at the time of the initial procedure with the option of staged revascularization; and (3) complete revascularization, in which immediate CABG or immediate multivessel PCI was performed for all major coronary lesions with a visually estimated diameter stenosis of ≥ 70% in vessels with reference vessel diameter^24^. Chronic total occlusions were also considered for recanalization. The primary outcome of the study was neurologically intact survival at 1-month defined as a Cerebral Performance Category score of 1–2. The secondary outcome was survival at 1-month.

### Statistical analysis

Continuous variables are presented as mean (standard deviation) or median (interquartile range) according to their distribution in the Kolmogorov–Smirnov test. Categorical variables are expressed as absolute number (percentage). Comparisons of the demographic and clinical characteristics among the groups were performed using the Student’s *t*-test, one-way analysis of variance, Mann–Whitney *U*-test, or the Kruskal–Wallis test as appropriate for continuous variables, and the chi-square test for categorical variables. To minimize the effect of treatment selection bias and potential confounding factors between the complete group and the incomplete/no immediate revascularization strategy groups, we used the IPTW method, which provides unbiased estimates of the treatment effect by creating a pseudo-population in which the treatment is randomized^25^. We assigned each patient a stabilized weight for each of the clinically significant baseline characteristics. For patients in the complete revascularization group, the stabilized weight was calculated as the product of the marginal probability of receiving complete revascularization and the inverse of the propensity score. For patients in the incomplete or no immediate revascularization group, the stabilized weight was the product of the marginal probability of incomplete/no immediate revascularization and the inverse of (1-propensity score). The data with IPTW were assessed for group comparability using the standardized mean difference (Supplementary Table S2 online).

The effect of complete revascularization was evaluated using two methods: crude multivariable logistic analysis and analysis of data with IPTW. In the crude analysis, baseline characteristics and coronary angiographic findings were first examined using univariate logistic analysis (Supplementary Tables S1 and S3 online). Variables with an entry-level significance (p < 0.05) in univariable analysis, age, sex, disease extent, and revascularization strategy, were selected for multivariable logistic regression analysis, which was interpreted as the association between revascularization strategies and outcomes. The Hosmer–Lemeshow test was performed to test the goodness-of-fit of the logistic regression model. In the analyses of IPTW data, logistic or linear regression was performed and the resulting OR estimates were interpreted as a closer approximation to the causal effect of the complete revascularization. All reported p-values are two-sided, and those smaller than 0.05 were considered statistically significant. All statistical analyses were performed using SAS version 9.4 (SAS Institute Inc., Cary, NC, USA) and IBM SPSS Statistics for Windows, version 21.0 (IBM Corp., Armonk, NY, USA).

## Supplementary Information

Supplementary Tables.

## Data Availability

The datasets generated during and/or analysed during the current study are available from the corresponding author on reasonable request.
