# Supplementary material for: Immediate complete revascularization showed better outcome in out-of-hospital cardiac arrest survivors with left main or triple-vessel coronary diseases
Source: Sci Rep. 2022 Mar 14;12:4354. doi: 10.1038/s41598-022-08383-x (PMC8921273; doi:10.1038/s41598-022-08383-x)
Supplement: Supplementary file 1 — Supplementary Tables. [file 41598_2022_8383_MOESM1_ESM.docx]

Supplementary Table S1. The clinical characteristics of patients in the complete revascularization group who received immediate coronary artery bypass grafting.

| Case number | Age/Sex | Comorbid disease | | | Previous stroke | Previous PCI | Arrest characteristics | | | | | GCS | STE on immediate ECG | Intervention before and during CAG | | Disease extent | Number of CTO | TTM | Neurologic outcome |
| --- | --- | --- | --- | --- | --- | --- | --- | --- | --- | --- | --- | --- | --- | --- | --- | --- | --- | --- | --- |
|  |  | HTN | DM | CKD |  |  | Witnessed | Bystander CPR | Initial shockable rhythm | No flow time, min | Total resuscitation duration, min |  |  | Vasopressor use | Extracorporeal life support |  |  |  |  |
| 1 | 54/M | N | Y | N | N | N | Y | Y | Y | 1 | 19 | 7 | N | N | N | 3VD | 1 | N | Good |
| 2 | 57/M | Y | Y | N | N | Y | Y | Y | N | 1 | 13 | 5 | Y | Y | N | 3VD | 2 | N | Good |
| 3 | 64/M | N | N | N | N | N | Y | Y | N | 0 | 28 | 3 | N | N | Y | LM+3VD | 0 | N | Good |
| 4 | 77/M | Y | Y | N | N | N | Y | Y | N | 0 | 2 | 4 | Y | Y | Y | LM+3VD | 2 | N | Good |
| 5 | 69/M | Y | Y | N | Y | N | Y | Y | N | 0 | 10 | 3 | Y | Y | N | 3VD | 2 | N | Poor |

CAG, coronary angiography; CKD, Chronic kidney disease; CPR, cardiopulmonary resuscitation; CTO, Chronic total occlusion; DM, Diabetes Mellitus; ECG, electrocardiogram; GCS, Glasgow coma scale; HTN, Hypertension; LM, left main; PCI, percutaneous coronary intervention; STE, ST-segment elevation; TTM, Targeted temperature management; VD, vessel disease.

Supplementary Table S2. Baseline and clinical characteristics of patients after analysis by inverse probability of treatment weighting

| Characteristics | Complete revascularization | No immediate or incomplete revascularization | Standardized difference of mean values | *P*-value |
| --- | --- | --- | --- | --- |
| Age, years | 62.5 (9.45) | 63.8 (11.61) | 0.128 | 0.55 |
| Male sex | 25.0 (97.4) | 100.3 (86.0) | 0.420 | 0.06 |
| Comorbid disease |  |  |  |  |
| Hypertension | 15.5 (60.4) | 62.8 (53.8) | 0.132 | 0.59 |
| Diabetes mellitus | 12.0 (46.9) | 48.8 (41.9) | 0.101 | 0.69 |
| Chronic kidney disease | 0.8 (3.1) | 8.7 (7.4) | 0.194 | 0.39 |
| Previous stroke | 2.9 (11.2) | 11.7 (10.0) | 0.869 | 0.89 |
| Previous PCI | 3.0 (11.7) | 14.3 (12.2) | 0.774 | 0.96 |
| Arrest characteristics |  |  |  |  |
| Witnessed | 3.3 (13.0) | 19.5 (16.7) | 0.105 | 0.61 |
| Bystander CPR | 8.4 (32.9) | 43.7 (37.5) | 0.095 | 0.69 |
| Initial shockable rhythm | 13.8 (53.8) | 68.3 (58.5) | 0.095 | 0.70 |
| No flow time, min | 3.29 (4.64) | 3.54 (5.48) | 0.052 | 0.82 |
| Total resuscitation duration, min | 28.58 (25.24) | 30.91 (20.68) | 0.108 | 0.66 |
| ST-segment elevation on immediate ECG | 10.8 (42.2) | 55.9 (48.0) | 0.116 | 0.64 |
| Intervention before and during CAG |  |  |  |  |
| Vasopressor use | 14.2 (55.6) | 74.5 (63.9) | 0.169 | 0.50 |
| Extracorporeal life support | 17.7 (69.0) | 79.6 (68.3) | 0.015 | 0.95 |
| Disease extent |  |  | 0.090 | 0.92 |
| Left main artery only | 15.3 (59.9) | 73.3 (62.9) |  |  |
| Left main artery plus ≥1 vessel disease | 5.6 (21.8) | 21.3 (18.2) |  |  |
| Triple vessel disease | 4.7 (18.3) | 22.1 (18.9) |  |  |
| Chronic total occlusion ≥1 | 10.6 (41.2) | 54.9 (47.1) | 0.118 | 0.66 |
| Targeted temperature management | 14.9 (58.3) | 77.6 (66.5) | 0.171 | 0.49 |

Values are presented as mean (standard deviation), median (interquartile range), or number (percentage) as appropriate.

CAG, coronary angiography; CPR, cardiopulmonary resuscitation; ECG, electrocardiogram; PCI, percutaneous coronary intervention.

Supplementary Table S3. Univariable logistic regression analysis for 1-month good neurological outcome in the study patients.

| Characteristics | Odds Ratio | 95% confidence interval | *P*-value |
| --- | --- | --- | --- |
| Age, years | 0.953 | 0.924–0.984 | 0.003 |
| Male sex | 0.813 | 0.313–2.109 | 0.67 |
| Comorbid disease |  |  |  |
| Hypertension | 0.514 | 0.259–1.020 | 0.06 |
| Diabetes Mellitus | 0.480 | 0.234–0.987 | 0.05 |
| Chronic kidney disease | 0.200 | 0.025–1.625 | 0.13 |
| Previous stroke | 0.500 | 0.133–1.880 | 0.31 |
| Previous PCI | 0.660 | 0.224–1.948 | 0.45 |
| Arrest characteristics |  |  |  |
| Witnessed | 1.194 | 0.480–2.971 | 0.70 |
| Bystander CPR | 0.853 | 0.427–1.701 | 0.65 |
| Initial shockable rhythm | 2.059 | 1.011–4.191 | 0.05 |
| No flow time, min | 0.935 | 0.860–1.017 | 0.12 |
| Total resuscitation duration, min | 0.962 | 0.941–0.982 | <0.001 |
| ST-segment elevation on immediate ECG | 0.456 | 0.229–0.909 | 0.03 |
| Intervention before and during CAG |  |  |  |
| Vasopressor use | 0.452 | 0.225–0.907 | 0.03 |
| Extracorporeal life support | 0.441 | 0.202–0.964 | 0.04 |
| Disease extent |  |  |  |
| Triple vessel disease | Reference |  | 0.48 |
| Left main artery only | 3.100 | 0.492–19.549 | 0.23 |
| Left main artery plus ≥1 vessel disease | 1.063 | 0.519–2.176 | 0.87 |
| Chronic total occlusion ≥1 | 1.067 | 0.542–2.100 | 0.85 |
| Targeted temperature management | 0.955 | 0.466–1.960 | 0.90 |
| Revascularization strategy |  |  |  |
| No immediate | Reference |  | 0.03 |
| Incomplete | 1.625 | 0.673–3.923 | 0.28 |
| Complete | 3.904 | 1.413–10.785 | 0.009 |

CAG, coronary angiography; CPR, cardiopulmonary resuscitation; ECG, electrocardiogram; PCI, percutaneous coronary intervention.

Supplementary Table S4. Univariable logistic regression analysis for 1-month survival in the study patients.

| Characteristics | Odds Ratio | 95% confidence interval | *P*-value |
| --- | --- | --- | --- |
| Age, years | 0.935 | 0.905–0.966 | <0.001 |
| Male sex | 0.716 | 0.282–1.816 | 0.48 |
| Comorbid disease |  |  |  |
| Hypertension | 0.494 | 0.257–0.949 | 0.03 |
| Diabetes Mellitus | 0.542 | 0.280–1.048 | 0.07 |
| Chronic kidney disease | 0.405 | 0.101–1.629 | 0.20 |
| Previous stroke | 0.366 | 0.109–1.225 | 0.10 |
| Previous PCI | 0.695 | 0.263–1.838 | 0.46 |
| Arrest characteristics |  |  |  |
| Witnessed | 0.567 | 0.239–1.348 | 0.20 |
| Bystander CPR | 0.713 | 0.369–1.379 | 0.32 |
| Initial shockable rhythm | 1.000 | 0.524–1.910 | >0.999 |
| No flow time, min | 0.991 | 0.934–1.052 | 0.78 |
| Total resuscitation duration, min | 0.973 | 0.957–0.990 | 0.001 |
| ST-segment elevation on immediate EKG | 0.524 | 0.274–1.002 | 0.05 |
| Intervention before and during CAG |  |  |  |
| Vasopressor use | 0.416 | 0.210–0.824 | 0.01 |
| Extracorporeal life support | 0.448 | 0.222–0.904 | 0.03 |
| Disease extent |  |  |  |
| Triple vessel disease | Reference |  | 0.81 |
| Left main artery only | 1.436 | 0.229–9.000 | 0.70 |
| Left main artery plus ≥1 vessel disease | 0.855 | 0.435–1.682 | 0.65 |
| Chronic total occlusion ≥1 | 1.458 | 0.765–2.779 | 0.25 |
| Targeted temperature management | 1.734 | 0.868–3.461 | 0.12 |
| Revascularization strategy |  |  |  |
| No immediate | Reference |  | 0.23 |
| Incomplete | 1.285 | 0.596–2.771 | 0.28 |
| Complete | 2.255 | 0.871–5.839 | 0.09 |

CAG, coronary angiography; CPR, cardiopulmonary resuscitation; ECG, electrocardiogram; PCI, percutaneous coronary intervention.
